# Polymer Adhesin Domains in Gram-Positive Cell Surface Proteins

**DOI:** 10.3389/fmicb.2020.599899

**Published:** 2020-11-26

**Authors:** Michael A. Järvå, Helmut Hirt, Gary M. Dunny, Ronnie P.-A. Berntsson

**Affiliations:** ^1^Department of Medical Biochemistry and Biophysics, Umeå University, Umeå, Sweden; ^2^Department of Microbiology and Immunology, University of Minnesota, Minneapolis, MN, United States; ^3^Wallenberg Centre for Molecular Medicine, Umeå University, Umeå, Sweden

**Keywords:** adhesin, Gram-positive, conjugation, biofilm, binding

## Abstract

Surface proteins in Gram-positive bacteria are often involved in biofilm formation, host-cell interactions, and surface attachment. Here we review a protein module found in surface proteins that are often encoded on various mobile genetic elements like conjugative plasmids. This module binds to different types of polymers like DNA, lipoteichoic acid and glucans, and is here termed *polymer adhesin domain.* We analyze all proteins that contain a polymer adhesin domain and classify the proteins into distinct classes based on phylogenetic and protein domain analysis. Protein function and ligand binding show class specificity, information that will be useful in determining the function of the large number of so far uncharacterized proteins containing a polymer adhesin domain.

## Bacterial Adhesion in Gram-Positive Bacteria

Bacteria colonize host tissues by adhering to specific surfaces and by establishing bacterial biofilm communities. In Gram-positive bacteria this is often mediated by surface proteins that are anchored to the cell-wall through a sortase-dependent LPxTG-motif (Geoghegan and Foster, 2015; Foster, 2019). Pili and fimbriae are well known examples that form micrometer long filaments that protrude out from the cell allowing easy attachment to targets (Kang and Baker, 2012; Lukaszczyk et al., 2019). Other classes of adhesion proteins exist such as microbial surface components recognizing adhesive matrix molecules (MSCRAMMs), near iron transporter (NEAT) motif family, tandemly repeated three-helical bundles, G5-E domain repeat family, and legume-lectin-like cadherin-like family. For these classes there are multiple thorough reviews and book chapters detailing their structural and functional properties (Foster et al., 2014; Geoghegan and Foster, 2015; Foster, 2019; Dufrêne and Viljoen, 2020). In general, these proteins are comprised of a C-terminal stalk region built from heavily glycosylated disordered regions, coiled-coils, or tandem domain repeats. The N-terminus often feature one or several adhesion modules that are key to the functional part of the protein, and the stalk projects this region away from the peptidoglycan cell-wall, allowing access to the extracellular environment. They interact with key components in the extracellular matrix of their host to facilitate one or several types of pathogenic mechanisms such as surface attachment, host cell internalization, biofilm formation, immune evasion, and/or bactericide/antibiotic resistance.

However, a family of adhesion proteins found in lactic acid bacteria (*Lactobacillales*) cannot readily be categorized into any of the above-mentioned families. In *Streptococcus* these proteins go by the names Glucan-binding protein C, Dextran-binding lectin, and Antigen I/II, and in *Lactococcus* and *Enterococcus* they are called Aggregation substance. In *Streptococcus* these adhesion proteins promote cariogenicity through attachment to tooth surfaces (Jenkinson and Demuth, 1997; Sato et al., 2002a; Lynch et al., 2013). In *Enterococcus* and *Lactococcus* they are found on conjugative plasmids where they facilitate mating pair formation (Hirt et al., 2000; Waters and Dunny, 2001; Waters et al., 2003; Chuang et al., 2009; Schmitt et al., 2018). Apart from surface adherence and bacterial aggregation these proteins all feature a specific adhesion domain at their N-terminus, which most often is called Glucan-binding domain, Variable domain or Adhesion domain. Despite many similarities between these proteins no studies have yet systematically compared their functions and mechanisms.

As we will discuss in this review, the conserved adhesion module consistently adheres to various types of polymers associated with the extracellular matrix, such as collagen (Love et al., 1997; Holmes et al., 1998; Heddle et al., 2003), extracellular DNA (eDNA; Kohler et al., 2018; Schmitt et al., 2018), lipoteichoic acid (LTA; Waters et al., 2004; Schmitt et al., 2018), or different types of glucans (Sato et al., 2002b; Tamura et al., 2014; Mieher et al., 2018). Therefore, we will throughout this review refer to this adhesion module as the *polymer adhesin domain*.

## Domain Architecture and Function of Proteins With a Polymer Adhesin Domain

In order to gather a diverse population of polymer adhesin-containing proteins, we utilized the InterPro database (Mitchell et al., 2019). InterPro combines information from numerous other databases which uses various models such as hidden Markov models, scoring matrices, regular expressions, or other profiles that make up identifiable signatures to classify protein families. We gathered all entries that contained an N-proximal polymer adhesin domain (IPR013574) and a C-terminal LPxTG-motif (IPR019931). After removing outdated, deprecated, and fragmented entries the resulting list of 518 proteins was manually cross-referenced for associated literature, which gave us a list of proteins that had, to some extent, been functionally characterized (Table 1). One limitation with this approach is that the InterPro database relies on entries in UNIPROT. Thus it does not contain all known sequences in other databases, such as NCBI. In fact, we encountered two proteins in the literature that was not originally captured by the InterPro search. However, the advantage of using it is that all entries are curated. The final list thus corresponds to 21 proteins (Table 1).

**TABLE 1:** Proteins containing a polymer adhesin domain currently described in the literature and their associated meta data and references.

Class	Protein name	Gene(s)	PDB(s)	Accession code	Protein length	Organism	References
I	Glucan-binding protein C (GbpC)	*gbpC*	5UQZ/6CAM	Q8DTF1	583	*Streptococcus mutans*	Sato et al., 1997, 2002a,b; Mieher et al., 2018
I	Glucan-binding protein C (GbpC)	*gbpC*		Q4W7G2	617	*Streptococcus macacae*	Okamoto-Shibayama et al., 2006
I	Dextran-binding lectin A (DblA)	*dblA*		G5EIN8	1093	*Streptococcus criceti*	Tamura et al., 2014
I	Dextran-binding lectin B (DblB)	*dblB*		G5EIN9	1717	*Streptococcus criceti*	Tamura et al., 2014
I	Dextran-binding lectin B (DblB)	*dblB*		A8QYL3/B5BNX9	1425	*Streptococcus sobrinus*	Sato et al., 2009
II	Streptococcal surface protein A (SspA)	*sspA*		Q54185	1575	*Streptococcus gordonii*	Demuth et al., 1996; Holmes et al., 1998; Egland et al., 2001; Heddle et al., 2003; Jakubovics et al., 2005a, b; Giomarelli et al., 2006; Nobbs et al., 2007
II	Streptococcal surface protein B (SspB)	*ssp5/sspB*	2WD6	P16952/Q54186	1500	*Streptococcus gordonii*	Demuth et al., 1988; Duan et al., 1994; Demuth et al., 1996; Holmes et al., 1998; Heddle et al., 2003; Giomarelli et al., 2006; Nobbs et al., 2007; Forsgren et al., 2009; Forsgren et al., 2010
II	Cell-surface protein antigen (SpaP,PA/PAc/P1)	*spaP*		P23504	1562	*Streptococcus mutans serotype c*	Koga et al., 1986; Kelly et al., 1990; Hajishengallis et al., 1994; Heim et al., 2014; Jakubovics et al., 2005b
II	Cell-surface protein antigen (SpaP,PA/PAc/P1)	*spaP, pa, pac*	3IPK/3IOX/1JMM	C9E3B4/A8R5D9/P11657	1566	*Streptococcus mutans*	Sommer et al., 1987; Oho et al., 1998; Troffer-Charlier et al., 2002; Sato et al., 2002a; Nakano et al., 2006; Busscher et al., 2007; Larson et al., 2010; Heim et al., 2014; Sullan et al., 2015
II	Cell-surface antigen I/II (SpaA)	*spaA*		P21979	1528	*Streptococcus sobrinus*	Kuykindoll and Holt, 1996
II	Cell-surface antigen I/II	*pas*	6E36	Q9KW51	1310	*Streptococcus intermedius*	Petersen et al., 2001; Jakubovics et al., 2005b
III	SAG_1283			A8D815	1631	*Streptococcus dysgalactiae*	Davies et al., 2009
III	Agglutinin receptor I/II			KGI30072.1	1646	*Streptococcus pneumoniae*	Antic et al., 2017
III	Glucan-binding protein C (GbpC)			OYL08640.1	1634	*Streptococcus pneumoniae B1599*	Antic et al., 2017
IV	Aggregation substance (AS)	*prgB, asa1, asp1, asc10*	6EVU/6GED	Q04112	1305	*Enterococcus faecalis (plasmid: pCF10)*	Kreft et al., 1992; Bensing and Dunny, 1993; Rakita et al., 1999; Vanek, 1999; Hirt et al., 2000; Wells et al., 2000; Waters and Dunny, 2001; Isenmann et al., 2002; Waters et al., 2003, 2004; Chuang et al., 2009; Chuang-Smith et al., 2010; Bhatty et al., 2015; Kohler et al., 2018; Schmitt et al., 2018
IV	Aggregation substance (AS)	*prgB, asa1, asp1, asc10*		P17953	1296	*Enterococcus faecalis (plasmid: pAD1/pTEF1)*	Galli et al., 1990, 1992; Chow et al., 1993; Süßmuth et al., 2000; Rozdzinski et al., 2001
V	Sex factor aggregation protein	*cluA*		Q48588	1243	*Lactococcus lactis*	Godon et al., 1994; Gasson et al., 1995; Stentz et al., 2004, 2006; Kojic et al., 2011; Lukić et al., 2012

**FIGURE 1:**
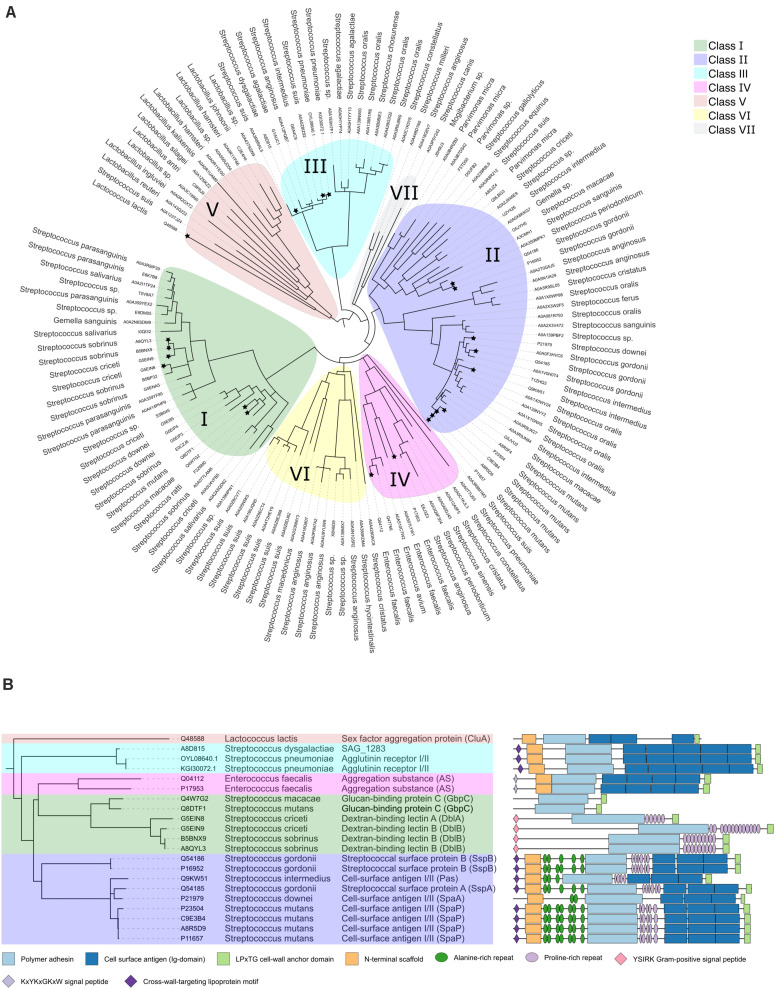
**(A)** Phylogenetic tree of a representative subset (<90% identity) of the polymer adhesin domain sequences as annotated in InterPro (Mitchell et al., 2019). Alignments done by ClustalO were used to calculate the phylogenetic relationships with PhyML (Dereeper et al., 2008) using 100 bootstraps. Visualization was done with iTol and brances with a bootstrap lower than 0.5 were collapsed. Each node is annotated with its respective UNIPROT accession code (in two instances refseq) and organism name. Clades are color coded, and nodes with associated literature are marked with a star. **(B)** Pruned tree highlighting the 21 reviewed protein entries, using the same class color coding as in panel **(A)**. Here the additional annotations include common protein name, and domain architecture as annotated by InterPro (Mitchell et al., 2019).

As we aimed to focus our review on the polymer adhesin domain we calculated a phylogenetic tree using only the polymer adhesin domain sequences rather than full-length protein. This was done to focus on the adhesive relationships of the polymer adhesins without the influence of large sequence and domain variety between the stalk regions. In this analysis we included all proteins with <90% sequence identity (and as low as 15%) of their polymer adhesin domain, plus all 21 reviewed proteins in Table 1, resulting in a phylogenetic tree with 131 proteins (Figure 1A). This phylogenetic analysis of only the polymer adhesin domain corresponded well with the predicted domain architecture of the 21 reviewed proteins (Figure 1B) and allowed us to divide them into five distinct classes (Classes I–V; Figure 1). The full phylogenetic analysis also indicates the presence of two additional classes (Classes VI and VII), containing proteins that so far lack any functional data in the literature (yellow and gray clades in Figure 1A).

Apart from the polymer adhesin domain and the LPxTG-motif, two other structural domains are found throughout the classes (except Class I proteins); (**i**) The C-terminal cell surface antigen domains (dark blue domain in Figure 1B), which are tandemly arranged bacterial immunoglobulins that often feature intramolecular isopeptide bonds and calcium-binding sites (Forsgren et al., 2010; Heim et al., 2014) and (**ii**) The N-terminal scaffold domain (orange domain in Figure 1B). It has been proposed that its function is to attach to its own C-terminal region to stabilize the structure and to make the polymer adhesin domain the most matrix exposed feature (Brady et al., 2010; Larson et al., 2010).

All known proteins belonging to these classes contain only one copy of the polymer adhesin domain. The polymer adhesin is often displayed quite a distance out from the cell wall, as there is usually either a coiled-coil stalk and/or one or more immunoglobulin domains between the polymer adhesin domain and the LPxTG cell-wall anchor. Based on the literature, we conclude that the polymer adhesin module likely exerts a core function in most of these proteins. The surrounding immunoglobulin domains likely act as helper modules to provide additional functionality or to display the polymer adhesin domain sufficiently far out from the cell surface. We wanted to investigate whether our classification of the polymer adhesin modules correlates with their variation in function, e.g., which ligands they bind and which pathogenic mechanisms they promote. To address this question, we went through the literature and looked at the available data for proteins associated with Classes I–V (as mentioned previously, Class VI and VII have no associated literature).

## Structure and Function of Polymer Adhesion Containing Proteins

**FIGURE 2:**
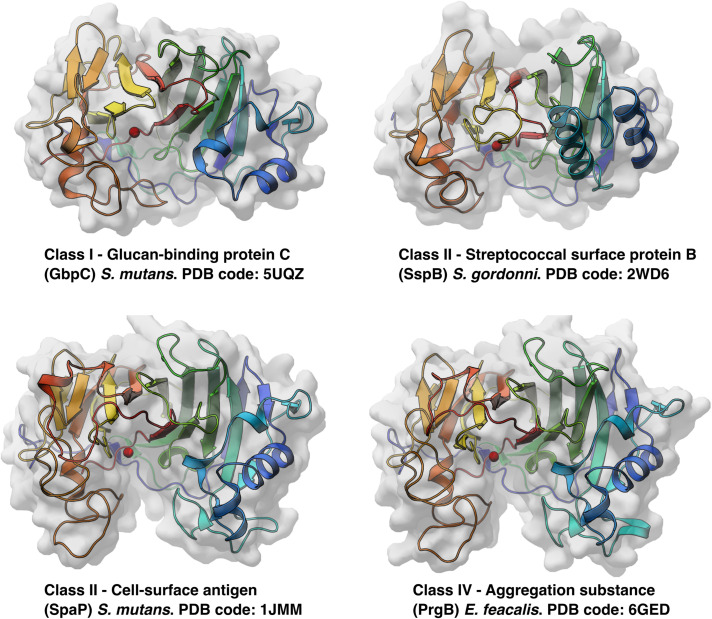
All available protein structures of unique proteins from three different classes of polymer adhesin containing proteins. Here drawn as cartoon representations, colored blue to red from the N-terminus and viewed from the same angle, the similarities in overall fold is seen, as well as their conserved cation binding site (red sphere) situated in the middle of the central ridge.

**FIGURE 3:**
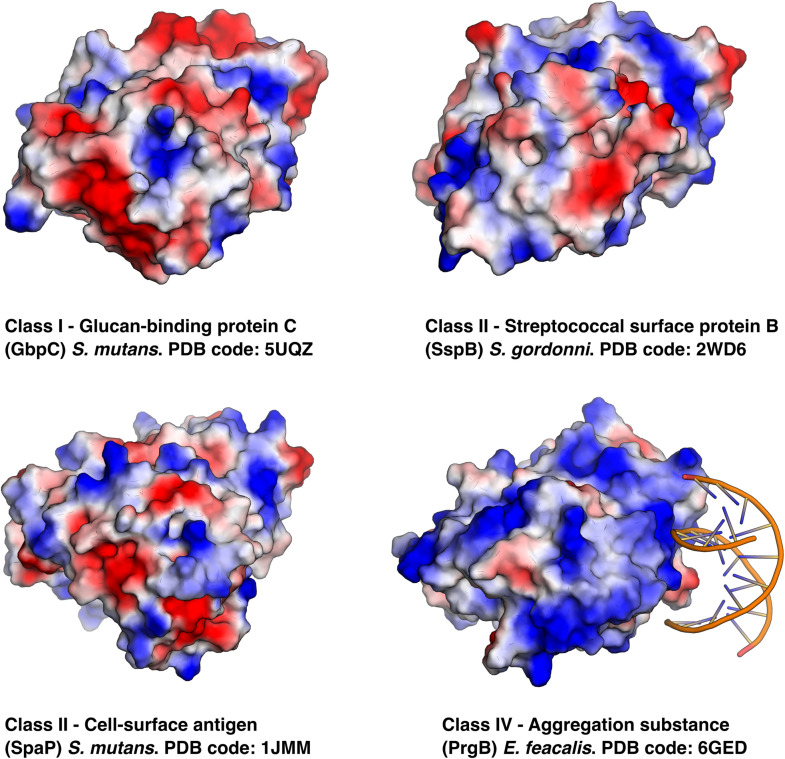
Same proteins as in Figure 2 but using electrostatic surface representations and in the case of PrgB its eDNA ligand is shown binding to the positively charged surface.

To date, there are six unique polymer adhesin domain structures deposited in the PDB, five of which are described in literature (Table 1). These structures originate from Classes I, II, and IV. Despite low sequence similarity (21–34%), the overall fold of the polymer adhesin domain remains remarkedly similar (Figure 2 and Table 2). In all structures, the core fold comprises an antiparallel beta-sandwich of 12–16 strands. On one side of this core, two lobes (made up of highly variable loops and short alpha-helices) create a central ridge. This ridge harbors a cation binding site that is conserved throughout the domain family (Figure 2). Due to the differences in primary sequence, the surface charge distribution of the domains also varies, with surfaces ranging from mostly negatively charged to mostly positively charged (Figure 3). None of these proteins have so far had their full-length structure determined, but the current structural evidence points toward that the N-terminal sequence preceding the polymer adhesin domain forms a coiled-coil with the sequence just C-terminal of said domain (Brady et al., 2010; Larson et al., 2010). This means that the tip of the proteins, thus the part extending furthest out from the cell wall, is the polymer adhesin domain.

**TABLE 2:**
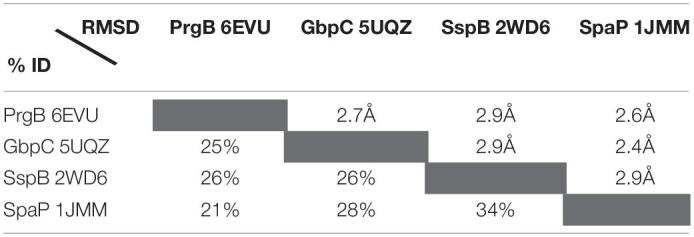
Sequence and r.m.s. deviations between the four structurally characterised polymer adhesin domains.

### Class I – Glucan-Binding Protein C and Dextran-Binding Lectin

This Class (green clade in Figure 1A) is divided into two subgroups: glucan-binding protein C (GbpC) and Dextran-binding lectin (Dbl) proteins. Counter-intuitively, both types bind dextran (branched, primarily α-1,6-glucans) whereas the Dextran-binding lectin proteins also bind to amylose (α-1,4-glucans) and to the α-1,3-branches on dextran (Okamoto-Shibayama et al., 2006). In contrast to the other classes, they do not contain any immunoglobulin domains at their C-terminus. Compared to GbpC, Dbl proteins have longer flanking coils and a long sequence insertion in the middle of the polymer adhesin domain (Figure 1). Dbls also feature an N-terminal YSIRK-motif (pink in Figure 1B). The YSIRK motif is unique to Streptococci and Staphylococci and enhances the efficiency of protein secretion (Bae and Schneewind, 2003) and enforce spatial positioning to the septal wall (Bierne and Dramsi, 2012).

The affinity of GbpC-polymer adhesins for various lengths of dextrans has been measured by isothermal calorimetry (ITC; Mieher et al., 2018). This enthalpy driven binding is stronger for longer polymers (highest reported affinity was ∼17 μM for a dextran with ∼390 glucose units). Based on these binding experiments, it was estimated that each GbpC-polymer adhesin unit adheres to 11–14 glucose units in a non-cooperative manner. Of the two determined GbpC structures, one is in a apo-state (PDB code: 5UQZ) whereas in the other (PDB code: 6CAM) two glucose molecules are modeled near the cation binding site (Mieher et al., 2018). Removal of a loop region overarching the cation binding site lead to a reduction, but not elimination, in glucose mediated biofilm formation (Mieher et al., 2018). As no other confirmatory experiments have been reported, the validity of this site being a glucan-binding site remains uncertain.

It has been shown that GbpC can interact with the host receptor salivary agglutinin (SAG) with nanomolar affinity and independently of SAGs glycosylation state (Purushotham and Deivanayagam, 2014; Mieher et al., 2018). SAG is also known as gp340 and is expressed from the “*deleted in membrane protein 1*” gene (DMBT1). It is a large extracellular matrix protein which features 13 repeats of the scavenger receptor cysteine rich domain 1 (iSRCR; Reichhardt et al., 2020). GbpC has been shown to interact with these iSRCR domains in a calcium-dependent fashion (Purushotham and Deivanayagam, 2014; Mieher et al., 2018), but since the fold of iSRCR is in itself calcium- dependent (Reichhardt et al., 2020) it is unknown whether or not the calcium-binding site of the polymer adhesin domain is involved in this interaction. Both the interaction to the entire SAG and the individual iSRCR domains are inhibited by the addition of dextran, which could indicate that they compete for the same binding site.

### Class II – Cell Surface Antigen I/II

Class II polymer adhesins (purple clade in Figure 1A) have been extensively studied for their prominent role in facilitating dental caries. The proteins that are found in this Class are usually named Agglutinin receptor I/II, as they were originally characterized by their ability to bind SAG (Demuth et al., 1988; Demuth et al., 1996). More recently Agglutinin receptor I/II are more commonly referred to as Cell-surface antigen I/II. The corresponding Class II proteins have been shown to interact with collagens (Love et al., 1997; Holmes et al., 1998; Heddle et al., 2003; Sciotti et al., 2006), fibrinogen (Brady et al., 2010), and laminin (Sciotti et al., 2006) – proteins characterized by long triple coiled-coils. These proteins have also been confirmed to bind to fibronectin (Giomarelli et al., 2006). All these proteins are common extracellular protein components that are often utilized by pathogens as an initial host interaction point (Vercellotti et al., 1985; Schwarz-Linek et al., 2006; Kang et al., 2013). It is mainly *S. mutans*, *S. oralis*, *S. sanguinis*, and *S. gordonii* that contain Class II proteins. *S. mutans* especially is a cause of dental caries (Hamada and Slade, 1980), whereas the other three species can act as opportunistic pathogens.

For three polymer adhesins in this Class the structure is known: SspB (*S. gordonii*; Forsgren et al., 2009), SpaP (*S. mutans*; Troffer-Charlier et al., 2002; Larson et al., 2010), and Pas (*S. intermedius*; Table 1). Pas has not been functionally characterized but is 85% identical to SpaP. As these proteins are similar, and it was known that sialic acid can inhibit SpaP binding to SAG (Demuth et al., 1990), it was thought that these proteins bind glucans. However, neither the SspB nor the SpaP polymer adhesin domains bind dextran (Mieher et al., 2018) and even though SspB was extensively tested on glycan arrays, no binding to any glycoconjugates has been observed (Forsgren et al., 2009). Despite the lack of direct interaction with glycans, the polymer adhesin in SpaP is important for both biofilm formation and dextran induced cellular aggregation (Mieher et al., 2018).

Furthermore, the Class II proteins SspB and SpaP can interact with the scavenger receptor cysteine-rich domain 1 (iSRCR) in a calcium-dependent fashion (Purushotham and Deivanayagam, 2014; Mieher et al., 2018) just as the Class I protein GbpC. In contrast to GbpC, however, this interaction is not inhibited by the addition of dextran. SspB and SpaP also have an additional independent iSRCR-interaction site (Larson et al., 2011). This site is located on the first two immunoglobulin domains, which the domain Class I proteins do not have. The second and third of these immunoglobulin domains on both SspB and SpaP bind calcium with submicromolar affinity (Duan et al., 1994; Forsgren et al., 2010; Larson et al., 2011; Nylander et al., 2011).

### Class III

Class III adhesins (cyan clade in Figure 1A) are most closely related to Class II adhesins in domain architecture (Figure 1B). Although not identified as such in Interpro, they do seem to feature similar alanine-rich and proline-rich repeats prior to, and after, the polymer adhesin domain as well. They also contain five Ig-domains rather than the three seen in Class II adhesins. Class III adhesins are found in *Streptococcus* species frequent in the upper respiratory tract of pigs (*S. suis*) and humans (*S. pneumoniae*), and in *S. agalactiae*, which can colonize the intestinal and vaginal microbiota (Barcaite et al., 2008). Two proteins have been shown to increase *S. pneumoniae* adhesion to ocular epithelia (Antic et al., 2017), but that remains the full extent of the known functions of this Class of adhesins. It is interesting to note, however, that bioinformatics analysis of Class III proteins have revealed that they are the result of horizontal gene transfer and that they are found on multiple mobile genetic elements (Davies et al., 2009).

### Class IV – Aggregation Substance

Class IV proteins (pink clade in Figure 1A) are mostly found in *Enterococcus faecalis* where they are found in sex pheromone responsive conjugative plasmids. Here they facilitate horizontal gene transfer via Type 4 Secretion Systems. Although they are mostly connected to Enterococci, they can spread to other species via conjugation. One protein in this Class has been structurally studied, namely PrgB (Aggregation substance) from the conjugative plasmid pCF10 (Schmitt et al., 2018). PrgB is one of the surface proteins expressed from the *prgQ* operon that encodes for all genes that are needed for conjugation. PrgB aids conjugation via surface attachment, biofilm formation, and cellular mating pair formation (Dunny et al., 1978; Bensing and Dunny, 1993; Bhatty et al., 2015; Schmitt et al., 2018). PrgB-like proteins are encoded by many other T4SS bearing plasmids in Enterococci, such as pAD1 (Galli et al., 1990, 1992; Chow et al., 1993; Süßmuth et al., 2000; Rozdzinski et al., 2001) and pD1 (Schmitt et al., 2020).

Expression of PrgB leads to cellular clumping (Dunny et al., 1978; Bhatty et al., 2015), which is dependent on the polymer adhesin domain binding to eDNA (Kohler et al., 2018; Schmitt et al., 2018). Surprisingly, this interaction does not take place at the conserved ridge with the cation binding site (Figure 2). Instead, eDNA binds in a sequence unspecific manner via charge interactions with surface exposed lysines and arginines (Schmitt et al., 2018; Figure 3). The same site also binds the core component of the Gram-positive cell-wall, LTA. As LTA is mainly composed of repeating units of ribitol or glycerol phosphate, it has a similar charge distribution to DNA. Compared to other polymer adhesin domain structures, the surface of the domain in PrgB is positively charged (Figure 3), enabling it to bind the negatively charged eDNA and LTA. The ability of PrgB to induce cellular aggregation and facilitate biofilm formation is completely dependent on the polymer adhesin domain, as deletion of this domain completely abrogates these functions (Bhatty et al., 2015; Schmitt et al., 2018). In contrast, when all domains between the polymer adhesin domain and the C-terminal LPxTG motif are removed cell aggregation and binding to LTA are still observed (Waters et al., 2004). Like Class II adhesins, PrgB promote adherence to fibronectin (Rozdzinski et al., 2001; Isenmann et al., 2002) and fibrinogen through its polymer adhesin domain (Chuang et al., 2009). Using in-frame deletions it has also been shown that the polymer adhesin domain is responsible for adhering to macrophages (Süßmuth et al., 2000). PrgB and its homologs are strong virulence factors in various infection models, including *C. elegans* (Bhatty et al., 2015) and rabbit endocarditis (Chow et al., 1993; Schlievert et al., 1998), where they play an important role in both vegetation formation and pathogenicity.

### Class V – CluA-Like Aggregation Substance

Only one protein in Class V (salmon clade in Figure 1A) has been functionally characterized, namely CluA from *Lactococcus lactis*. CluA is functionally homologous to the Class IV protein PrgB, performing highly similar functions related to cellular aggregation and conjugation (Godon et al., 1994; Stentz et al., 2004). The polymer adhesin domain of CluA has not been explicitly studied, but due to the overall similarity in effect of CluA compared to PrgB, it is likely that the polymer adhesin of CluA works in a similar manner by adhering to cell-wall components and host surfaces.

### Class VI and VII

We completely lack studies on any protein from Class VI or VII (yellow and gray clades, respectively, in Figure 1A). Domain analysis of proteins from these two classes reveal that they contain mucin (or mucin-like) binding protein domains, indicating that these proteins could be involved in binding to molecules associated with the extracellular matrix.

## Protein Functionalities Span the Classes

### Polymer-Binding Induced Aggregation

The most striking common denominator between the different classes of polymer adhesins is their propensity to bind specific types of polymers; Class I directly interact with positively charged oligosaccharides such as dextran (GbpC) and glucans (Dbl); Class II interact with an array of host proteins with coiled-coil features such as collagen, fibrinogen, and laminin; Class IV bind negatively charged polymers such as eDNA and LTA.

The specific binding site of these polymers has remained largely unknown until the eDNA binding site was elucidated for PrgB recently (Schmitt et al., 2018). Somewhat surprisingly, the PrgB polymer adhesin domain binds DNA on the opposite side of the ridge with the cation binding site (Figure 3), which was the proposed glycan binding site. For GbpC, the current literature suggests that the GbpC polymer adhesin domain binds dextran in the cleft containing the cation binding site (Mieher et al., 2018). However, based on the available data of the polymer adhesin domains, we hypothesize a different mechanism for polymer binding by these proteins. In this model, the polymer adhesin domains bind their target polymers via their surface, like the eDNA and LTA binding in PrgB. The driving factor in this adhesion is the avidity effects that naturally occur when polymers accumulate, which has been demonstrated for both GbpC and PrgB (Schmitt et al., 2018).

### Post-translational Protease Processing

Several of the polymer adhesin domain containing proteins are known to be post-translationally processed. PrgB from Class IV, is post-translationally cleaved in the unstructured region between the polymer adhesin-domain and the following immunoglobulin module (Nakayama et al., 1992; Schmitt et al., 2020). This has been linked to another surface protein, PrgA, which is expressed from the same operon (Hirt et al., 2000; Bhatty et al., 2015; Schmitt et al., 2020). The polymer adhesion domain of SpaP from Class II is also known to be enzymatically released from the cell surface (Russell et al., 1980; Sommer et al., 1987), thereby changing the cell’s hydrophobicity properties and facilitating biofilm release (Lee, 1992; Lee et al., 1996). Whether this enzymatic release is specific to these two proteins or something that is more common throughout the classes is not yet known. It does point to these proteins being actively degraded to facilitate involved biofilm dispersion, a poorly understood key phase of the biofilm life cycle (Rumbaugh and Sauer, 2020). An analogous feature can be observed in the well-studied Gram-negative RTX-adhesins, which are proteolytically released in response to nutrient restriction (Guo et al., 2019).

### Host Receptor Interactions

As these polymer adhesin-containing proteins are cell-wall anchored and surface exposed, they are often used in the interaction with host receptors. As mentioned previously, the polymer adhesin domains from GbpC (Class I), SspB and SpaP (Class II) have strong affinity for SAG and its subdomain iSRCR in a calcium-dependent fashion (Purushotham and Deivanayagam, 2014; Mieher et al., 2018). Whether or not other classes also bind SAG is unknown as this has not been tested. Class II proteins interact with collagen type I, fibrinogen, laminin, and fibronectin (Love et al., 1997; Holmes et al., 1998; Heddle et al., 2003; Giomarelli et al., 2006; Sciotti et al., 2006; Brady et al., 2010). Similarly, *in vivo* studies suggest that PrgB promote adhesion to fibrinogen, fibronectin, thrombospondin, vitronectin, and collagen type I (Rozdzinski et al., 2001; Isenmann et al., 2002). These extracellular matrix proteins are common targets for bacterial adhesion mechanisms (fibronectin in particular) and are thought to be a key component in establishing bacterial infections. However, the exact nature of the interactions of polymer adhesin domain containing proteins and various host receptors can vary widely and exact binding sites have not been established.

### A Concluding Model

**FIGURE 4:**
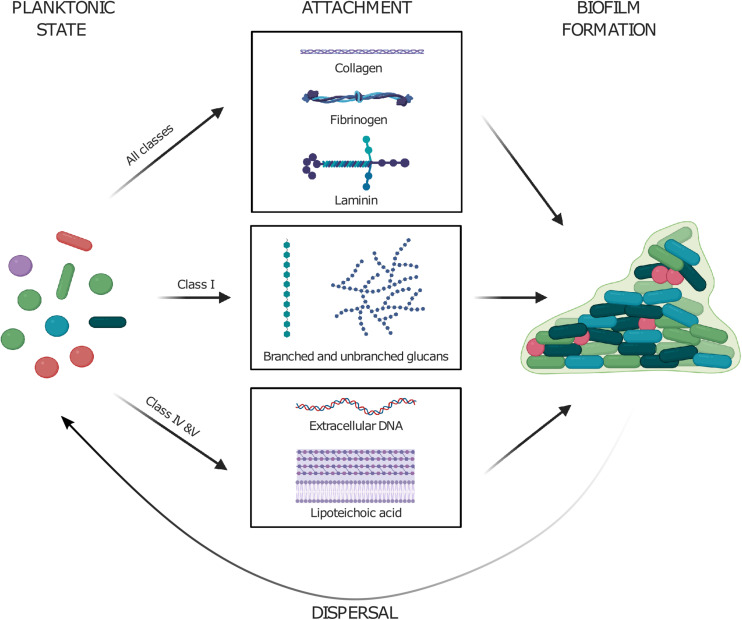
Planktonic cells utilize their polymer adhesin domain to attach to various surfaces by binding various molecules such as (i) coiled-coil proteins in extracellular matrix, (ii) polysaccharides, and (iii) negatively charged polymers such as eDNA and lipoteichoic acid. The attachment to these further drives the formation of cellular biofilms. Enzymatic cleavage of the polymer adhesin domain might aid in the dispersal of cells from mature biofilms. Created with BioRender.

Each class of polymer adhesin containing protein has developed its own niche and affinity toward different types of ligands, but the overall purpose of its existence appears to be largely conserved. We propose an overall model for polymer adhesin-contribution to life cycle of cellular biofilms in Figure 4, where they are involved in transitioning between the phases. In the transition from planktonic cells to attachment, different types of extracellular matrix molecules are recognized. GbpC and Dbl recognize dextran, amylose, and SAG. Antigen I/II recognize SAG and common subendothelial matrix proteins such as fibrinogen. Aggregation substance recognize negatively charged polymers (eDNA and LTA), as well as fibrinogen and similar matrix proteins. Because the polymer adhesion is avidity driven, initial attachment lead to further aggregation as the concentration of polymers and cells continue to increase and promote biofilm formation. They also actively recruit polysaccharides and eDNA which are two major components of bacterial biofilm. Finally, we hypothesize that proteolytic cleavage of the polymer adhesin domain might aid in cell dispersal events in mature biofilms.

## Outlook

Albeit more than 518 proteins contain a polymer adhesin domain, we conclude that this family of bacterial adhesins is relatively poorly characterized, since only 21 polymer adhesion domain containing proteins from 5 of the 7 classes have been studied functionally (Table 1). Interestingly, we found that these 21 studied proteins naturally fall into the same five separate classes whether they are clustered by polymer adhesin domain sequence identity or by the domain organization of the full-length protein (Figure 1B). Furthermore, the type of polymer that the proteins bind might correlate to the different classes, with Class I binding (positively charged) glucans, Class II mainly interacting with coiled-coil proteins and Class IV binding (negatively charged) eDNA and LTA. However, the ligand preferences of the polymer adhesin domain of the other classes have not been conclusively determined to date, so we cannot conclude that each class binds its own kind of polymer.

Class IV PrgB is one of the better studied proteins with a cell-wall anchor and a polymer adhesion domain. However, there are many more proteins with these same two properties that are also encoded from genes on conjugative plasmids in bacilli, plasmids that most often also encode for antibiotic resistance. Like their characterized counterparts, these proteins are very likely to be involved in biofilm formation and adhesion to specific host receptors, and therefore probably also strong virulence factors. Studying these proteins will be useful to further understand virulence and the mechanism of adhesion processes in Gram-positive bacteria.

## Author Contributions

MJ: conceptualization, performed analysis, and writing the manuscript. HH: conceptualization and validation. GD: conceptualization and aided in writing. RB: conceptualization, analysis, and writing. All authors contributed to the article and approved the submitted version.

## Conflict of Interest

The authors declare that the research was conducted in the absence of any commercial or financial relationships that could be construed as a potential conflict of interest.
